# Disulfiram Acts as a Potent Radio-Chemo Sensitizer in Head and Neck Squamous Cell Carcinoma Cell Lines and Transplanted Xenografts

**DOI:** 10.3390/cells10030517

**Published:** 2021-02-28

**Authors:** Wenhao Yao, Xu Qian, Sebastian Ochsenreither, Ferrone Soldano, Albert B. DeLeo, Holger Sudhoff, Felix Oppel, Andreas Kuppig, Konrad Klinghammer, Andreas M. Kaufmann, Andreas E. Albers

**Affiliations:** 1 Department of Otolaryngology, Head and Neck Surgery, Charité-Universitätsmedizin Berlin, Freie Universität Berlin andHumboldt-Universität zu Berlin, Berlin Institute of Health, Charité Campus Benjamin Franklin, Hindenburgdamm 30, 12203 Berlin, Germany; wenhao.yao@hotmail.com; 2 Department of Otolaryngology & Head and Neck Surgery, Ruijin Hospital, School of Medicine, Shanghai Jiao Tong University, Shanghai 200025, China; 3 Department of Clinical Laboratory, Institute of Cancer and Basic Medicine (ICBM), Chinese Academy of Sciences, Cancer Hospital of the University of Chinese Academy of Sciences (Zhejiang Cancer Hospital), Hangzhou 310015, China; Qianxu@zjcc.org.cn; 4 Department of Hematology, Oncology and Tumor Immunology, Charité-Universitätsmedizin Berlin, Freie Universität Berlin and Humboldt-Universität zu Berlin, Berlin Institute of Health, Charité Campus Benjamin Franklin, Hindenburgdamm 30, 12203 Berlin, Germany; sebastian.ochsenreither@charite.de (S.O.); konrad.klinghammer@charite.de (K.K.); 5 Department of Surgery, Massachussets General Hospital, Harvard Medical School, Boston, MA 02138, USA; SFERRONE@mgh.harvard.edu; 6 Cancer Institute, University of Pittsburgh, Pittsburgh, PA 15106, USA; deleoab@gmail.com; 7 Department of Pathology, University of Pittburgh, Pittburgh, PA 15106, USA; 8 Department of Otolaryngology, Head and Neck Surgery, Klinikum Bielefeld, Teutoburger Str. 50, 33604 Bielefeld, Germany; HOLGER.SUDHOFF@klinikumbielefeld.de (H.S.); FELIX.OPPEL@klinikumbielefeld.de (F.O.); 9 Department of Radiation oncology and Radiotherapy, Charité-Universitätsmedizin Berlin, Freie Universität Berlin and Humboldt-Universität zu Berlin, Berlin Institute of Health, Charité Campus Benjamin Franklin, Hindenburgdamm 30, 12203 Berlin, Germany; andreas.kuppig@charite.de; 10 Clinic for Gynecology, Charité-Universitätsmedizin Berlin, Freie Universität Berlin and Humboldt-Universität zu Berlin, Berlin Institute of Health, Charité Campus Virchow Klinikum, Augustenburger Platz 1, 13353 Berlin, Germany; andreas.kaufmann@charite.de

**Keywords:** disulfiram, HNSCC stem cells, aldehyde dehydrogenase, cisplatin, irradiation

## Abstract

The poor prognosis of locally advanced and metastatic head and neck squamous cell carcinoma (HNSCC) is primarily mediated by the functional properties of cancer stem cells (CSCs) and resistance to chemoradiotherapy. We investigated whether the aldehyde dehydrogenase (ALDH) inhibitor disulfiram (DSF) can enhance the sensitivity of therapy. Cell viability was assessed by the 1-(4,5-dimethylthiazol-2-yl)-3,5-diphenylformazan (MTT) and apoptosis assays, and the cell cycle and reactive oxygen species (ROS) levels were evaluated by fluorescence-activated cell sorting (FACS). The radio-sensitizing effect was measured by a colony formation assay. The synergistic effects were calculated by combination index (CI) analyses. The DSF and DSF/Cu^2+^ inhibited the cell proliferation (inhibitory concentration 50 (IC_50_) of DSF and DSF/Cu^2+^ were 13.96 μM and 0.24 μM). DSF and cisplatin displayed a synergistic effect (CI values were <1). DSF or DSF/Cu^2+^ abolished the cisplatin-induced G2/M arrest (from 52.9% to 40.7% and 41.1%), and combining irradiation (IR) with DSF or DSF/Cu^2+^ reduced the colony formation and attenuated the G2/M arrest (from 53.6% to 40.2% and 41.9%). The combination of cisplatin, DSF or DSF/Cu^2+^, and IR enhanced the radio-chemo sensitivity by inducing apoptosis (42.04% and 32.21%) and ROS activity (46.3% and 37.4%). DSF and DSF/Cu^2+^ enhanced the sensitivity of HNSCC to cisplatin and IR. Confirming the initial data from patient-derived tumor xenograft (PDX) supported a strong rationale to repurpose DSF as a radio-chemosensitizer and to assess its therapeutic potential in a clinical setting.

## 1. Introduction

Head and neck squamous cell carcinoma (HNSCC) is the sixth-most common cancer, accounting for over 600,000 new cases and 350,000 deaths worldwide per year [1]. Although HNSCC is highly curable at early stages, about 60% of HNSCC patients are diagnosed with the locoregionally advanced disease (stage III or IV), which is associated with poor survival. Local therapies, such as surgery, followed by radiation therapy (RT) with or without concomitant chemotherapy (CT), are the key components of the initial treatment of locally advanced (LA) HNSCC [2]. During the past decade, organ preservation protocols with chemoradiation have also been increasingly applied, where cisplatin-based chemotherapy is combined with concurrent locoregional radiotherapy [3]. Despite advances in the treatment of HNSCC, the survival rates have remained largely unchanged over the past 30 years, with a five-year survival rate of less than 50%. Treatment resistance, as well as tumor recurrence, remain critical drawbacks [4]. Therefore, there is a pressing need to improve the treatment efficacy and develop novel treatment strategies to improve the response to radio-chemotherapy and targeted therapies. 

Disulfiram (DSF), a hydrophobic and symmetrical molecule of the dithiocarbamate family, is available for oral administration and is approved to treat alcohol dependence [5]. DSF has a strong affinity to thiols and forms a covalent linkage with an active-site cysteine of aldehyde dehydrogenase (ALDH), inactivating the enzyme and leading to the accumulation of acetaldehyde, causing an aversion to alcohol [6]. Despite its multiple pharmacologic activities, a prolonged treatment with DSF has negligible and reversible adverse effects, and it is still considered a safe drug [7]. Particularly, DSF was recently found to have significant anticancer activity against a number of cancer types, such as melanoma [8], glioblastoma [9], prostate cancer [10], and breast cancer [11], both in vitro and in vivo. An epidemiological study showed that cancer-specific mortality was 34% higher among 3038 previous DSF users (who were prescribed DSF for alcohol dependency only before their cancer diagnosis) than among 1177 continuing users (who were prescribed DSF both before and after diagnosis) [12].

In cells, DSF converts to diethyldithiocarbamate (deDTC), and two molecules of deDTC bind to one molecule of Cu^2+^ to form the Cu [deDTC]_2_ complex (DSF/Cu^2+^) [13,14]. Cu^2+^ is an essential trace element for life, as it plays a crucial role in redox reactions and the generation of reactive oxygen species (ROS) in human cells [15]. The overall stoichiometry of the reaction with DSF:Cu^2+^ is a molar ratio of 1.0:0.9, which presumably could be used as a reference when DSF acts as a copper ionophore in the substance combination. It is likely that this may be the mechanism for the reaction of DSF with copper (II) ions under biological conditions [16]. The copper-binding activity of DSF may also be involved in this mode of action, because the formation of organic copper complexes appears to be responsible for proapoptotic proteasome inhibition [14]. The observed anticancer activities of DSF are the induction of oxidative stress, cytotoxicity due to acetaldehyde accumulation, and proteasome and nuclear factor-κB inhibition [17]. Other proteasome inhibitors, including deDTC, have chemo-radio-sensitizing effects, and this may also apply to DSF, further increasing its potential as an anticancer compound [18]. Notably, DSF and its metabolites could serve as broadly applicable and well-tolerated drugs for improving cancer treatment.

Cisplatin, one of the most widely used platinum agents, is the most common drug for combined CT/RT treatment in HNSCC. Biologically, cisplatin binds to DNA, resulting in adducts, and also favors the accumulation of intracellular free radicals [19]. However, many patients show an inherent resistance to cisplatin, which may consequently lead to an unfavorable treatment response. IR induces various DNA damage, but double-stranded breaks have the most cytotoxic effects, which can perturb the cell cycle progression at different stages, mainly inducing a G2/M phase arrest [20]. Moreover, even for advanced irradiation (IR) techniques and protocols, the control rates remain relatively low and unpredictable [21]. In practice, one of the major challenges in RT is the prediction of the tumor’s radio resistance in response to IR in order to optimize the given dose for the maximal tumor cytotoxic effect and minimal damage to normal tissues [22]. Following the treatment with cisplatin and IR, the cells were demonstrated to have a tendency to dwell longer in the G2/M phase, and the genomic integrity of the cells is maintained by activating the DNA repair processes before entering mitosis, which leads to increased cell survival, so that the release from this phase could be used to push the cells into apoptosis [23,24]. Efficient DNA repair in cancer cells is an important mechanism of therapy resistance, and therefore, the inhibition of DNA repair pathways would make cancer cells more sensitive to DNA-damaging agents like CT and RT [25]. 

To determine the potential of DSF in cancer therapy, it is important to dissect its various mechanisms of action and unravel the possible synergistic effects in combination with other therapeutic modalities. Here, we investigated a treatment strategy for DSF or DSF/Cu^2+^ in combination with cisplatin and IR against HNSCC cell lines in order to confirm their potent radio-chemo-sensitizing activity in vivo and chemo-sensitizing capability in vivo with a murine model employing xenotransplanted primary human HNSCC.

## 2. Materials and Methods

### 2.1. Cell Lines and Cell Culture

UM-SCC9, UM-SCC47, UMSCC11B (University of Michigan, Ann Arbor, MI, USA), and UT-SCC33 (University of Turku, Turku, Finland) were cultured in RPMI 1640 medium (Gibco, New York, NY, USA) supplemented with 10% fetal bovine serum (FBS) and 1% penicillin/streptomycin (Biochrom GmbH, Berlin, Germany). Cells were incubated at 37 °C in a humidified incubator with 95% air and 5% CO_2_ until 70–80% confluence. Cells were washed with phosphate-buffered saline (PBS) twice and detached with 0.5%/0.02% trypsin/EDTA (Ethylenediaminetetraacetate) solution (Biochrom, GmbH, Berlin, Germany). The reaction was stopped by adding the complete culture medium. After centrifugation at 200× *g* for 5 min, cells were prepared for the experiments or resuspended in a new culture medium for passage for future cultivation.

### 2.2. Reagents

DSF and copper (Sigma-Aldrich, St. Louis, MO, USA) were dissolved in dimethyl sulfoxide (DMSO; Sigma-Aldrich, Steinheim, Germany) and distilled water at a 10-mM stock concentration. Both of them were stored at −20 °C and freshly diluted to a working solution in the culture medium before use. We added DMSO solvent at an equivalent concentration in each control experimental group. The highest final concentration of DMSO in the viability experiments was 0.3%, which was low enough to not induce any effects, especially not apoptosis. The ratio of DSF and Cu^2+^ was set at 1:1 in all experiments.

### 2.3. 1-(4,5-Dimethylthiazol-2-yl)-3,5-Diphenylformazan (MTT) Cytotoxicity Assay 

Cells were seeded in 96-well plates at a density of 4000 cells/well in triplicates. The MTT assay was performed at the end of the 24–72-h incubation period. In brief, 10 μL of MTT labeling reagent were added to each well. After 4 h of incubation, 100 μL of solubilization solution was added to each well, and incubation was continued overnight. Optical density was measured using a Bio-Rad (Hercules, CA, USA) microplate reader at a 595-nm wavelength. The cell viability (%) data was averaged and normalized against the untreated control samples. 

### 2.4. Apoptosis Assessment

The apoptotic status was determined by an Annexin-V-FLUOS Staining Kit (Roche, Mannheim, Germany) using fluorescence-activated cell sorting (FACS; BD FACS Caliber Berlin, Germany) following the manufacturer’s protocol and analyzed by FlowJo V10 software (Becton Dickinson, Heidelberg, Germany). Cells were incubated in a 24-well plate at a density of 3 × 10^4^/mL for the indicated time intervals. Subsequently, cells were harvested and suspended at a density of 1 × 10^7^ cells/mL in 100-μL binding buffer containing 20-μL Annexin-V and 20-μL propidium iodide (PI) at room temperature in the dark for 15 min. Apoptosis and necrosis were evaluated using fluorescence channel 3 (FL3) (PI) and Fluorescence channel 1 (FL1) (Annexin-V). The percentage of cells was assessed in 4 quadrants: lower left (Annexin-V^−^/PI^−^) was presented for live cells, lower right (Annexin-V^+^/PI^−^) for early apoptosis, upper right (Annexin-V^+^/PI^+^) for late apoptosis, and upper left (Annexin-V^−^/PI^+^) for necrotic cells, respectively. 

### 2.5. Cell Cycle Analysis

Cells were exposed to various combinations of treatment for 48 h and subsequently harvested by trypsinization. After stopping the reaction with PBS plus 3% FBS, cells were fixed in 70% cold ethanol at 4 °C overnight. After two steps of PBS washing, samples were incubated with PI (1 mg/mL, Sigma, Darmstadt, Germany), RNase (10 mg/mL, Sigma, Darmstadt, Germany), and 1% Triton (Sigma-Aldrich, USA) for 30 min in the dark at room temperature. PI fluorescence of each sample was measured by flow cytometry with a 488-nm excitation wavelength, and the DNA content was analyzed using FlowJo software.

### 2.6. Measurement of Reactive Oxygen Species (ROS) Activity

Following 24 h of incubation with different drug combinations, cells were harvested and stained with MitoSOX Red Mitochondrial Superoxide Indicator (Molecular Probes/Invitrigen, Eugene, OR, USA) at 37 °C in a humidified incubator for 15 min. After incubation, samples were washed three times with PBS and analyzed by flow cytometry. Gated markers of ROS activity were set for the percentage of ROS activity in control cells incubated with MitoSOX reagent but without any drug treatment.

### 2.7. Irradiation (IR)

Cells were seeded in various treatment combinations 4 to 5 h before exposure to IR for dosages of 2, 4, 6, or 10 Gy using a medical linear accelerator (Clinac 600 C/D, Varian, Palo Alto, CA, USA) with a 6-MV (MeV, Million electron Volts) photon beam at 2.76 Gy/min. All samples were maintained in tissue culture flasks and positioned in a water tank for backscatter saturation of the beam to the flasks. An 8-cm-thick solid block of a water-equivalent material was put on the top of the plates to ensure that the photon dose was applied homogenously to the cells. Colony formation and ROS activity testing were performed 24 h after IR; cell cycle progression and apoptosis assessment were measured 48 h after IR.

### 2.8. Clonogenicity Survival Assay

Cells were incubated for an additional 24 h after IR. The added drug was completely removed by washing, and cells were reseeded in 6-well plates with fresh culture medium. After 9–12 days, the medium was removed, and colonies were washed with PBS twice before fixation by methanol and staining with 0.5% crystal violet. The number of colonies consisting of at least 50 cells was recorded. 

The survival fraction (SF) was calculated as follows: mean number of colonies counted/(number of cells inoculated *plating efficiency). Plating efficiency (PE) is defined as the mean number of colonies observed/number of cells inoculated for untreated controls. The cell survival curves were obtained by fitting into the linear-quadratic model (LQ model) using GraphPad Prism software (GraphPad Software Inc., San Diego, CA, USA) in formula Y = exp(−(a*x + b*(x^2^))). 

### 2.9. Spheroid Formation Assay

Cells were seeded in a cell culture flask onto a coated surface with 10% agarose in serum-free medium 263 (PAA, Laboratories GmbH, Heidelberg, Germany) supplemented with 10-ng/mL epidermal growth factor (EGF) and 10-ng/mL basic fibroblast growth factor (bFGF) (Biochrom, GmbH, Berlin, Germany). For passaging to at least the second generation or for the following experiments, spheroids were collected with a 40-μM mesh filter (Costar/Corning, Lowell, MA, USA) and were then dissociated into single cells by 5-min incubation with trypsin/EDTA at 37 °C in a water bath. The reaction was stopped by adding complete medium. Single cells were washed and plated into fresh culture medium under the same conditions.

For the spheroid formation assay, cells were treated with DSF or DSF/Cu^2+^ in 24-well ultra-low attachment plates (Corning, Costar, NY, USA) at the density of 2 × 104 cells/mL for 3 days and photographed at 50-fold magnification. Spheroids of 200 μm or more in diameter were counted. 

### 2.10. In Vivo Evaluation

The patient-derived tumor xenograft (PDX) models were established at EPO Berlin-Buch GmbH (Berlin, Germany) and propagated subcutaneously in NMRI (*nu/nu*) mice. EPO is fully accredited by the AAALAC (Association of Assessment and Accreditation of Laboratory Animal Care). For transplantation, the tumor was harvested and cut into small fragments. This study was approved by the local Institutional Review Board of Charité University Medicine, Berlin, Germany (EA4/019/12). All animal experiments were carried out in accordance with the United Kingdom coordinating committee on cancer research regulations for the welfare of animals and the German Animal Protection Law and were also approved by the local responsible authorities (LaGeSoBerlin, A0452/08).

The in vivo efficacy of disulfiram as a monotherapy and in combination with cisplatin was assessed in 3 PDX models of HNSCC using a setting with one mouse per model per arm. Tumor fragments of similar sizes (2 × 2 mm) were transplanted subcutaneously to NMRI *nu/nu* mice. At a palpable tumor size of 200 mm^3^, the mice (*n* = 1 to 2 per group) were treated with disulfiram (60 mg/kg/s.c.(subcutaneous injection)) three times a week and/or cisplatin (8 mg/kg, i.v. (intravenous injection)) once a week (Q7D). Mice in the vehicle group received 0.9% NaCl. Tumor growth was monitored two times a week by measuring the tumor volume [(width^2^ × length)/2] using a caliper. Treatment was continued over a period of 3 weeks, unless tumor size exceeded 2000 mm^3^ or animals showed a loss of body weight over 10%. Tumor volumes at the end of treatment compared to the beginning were used as the sensitivity parameter. Relative tumor volume (RTV) resembles tumor growth over the course of treatment (relative tumor volume in comparison to the timepoint of treatment initiation).

### 2.11. Statistical Analysis and Calculation of Drug Interactions

Statistical software GraphPad Prism 5.0 (GraphPad Software Inc., San Diego, CA, USA) was used for all statistical analyses. Values represent the mean ± standard deviation (SD) of at least duplicated wells in at least three independent experiments. Figures show results of one out of three representative experiments. Comparisons among multiple groups were performed using the one-way ANOVA test. A probability level of * *p* < 0.05 was recognized as statistically significant. 

The combination index (CI) method according to Chou-Talalay was applied to evaluate the drug interactions, which are based on the multiple drug effect equation derived from the median-effect principle of the mass-action law [26]. It provided quantitative determination for synergism (CI < 1), the additive effect (CI = 1), and antagonism (CI > 1) and established the algorithm using CompuSyn software (CompuSyn Inc., New York, NY, USA) for the automated simulation of drug combinations.

## 3. Results

### 3.1. DSF Inhibits Cells Proliferation in a Dose- and Time-Dependent Manner

**Figure 1:**
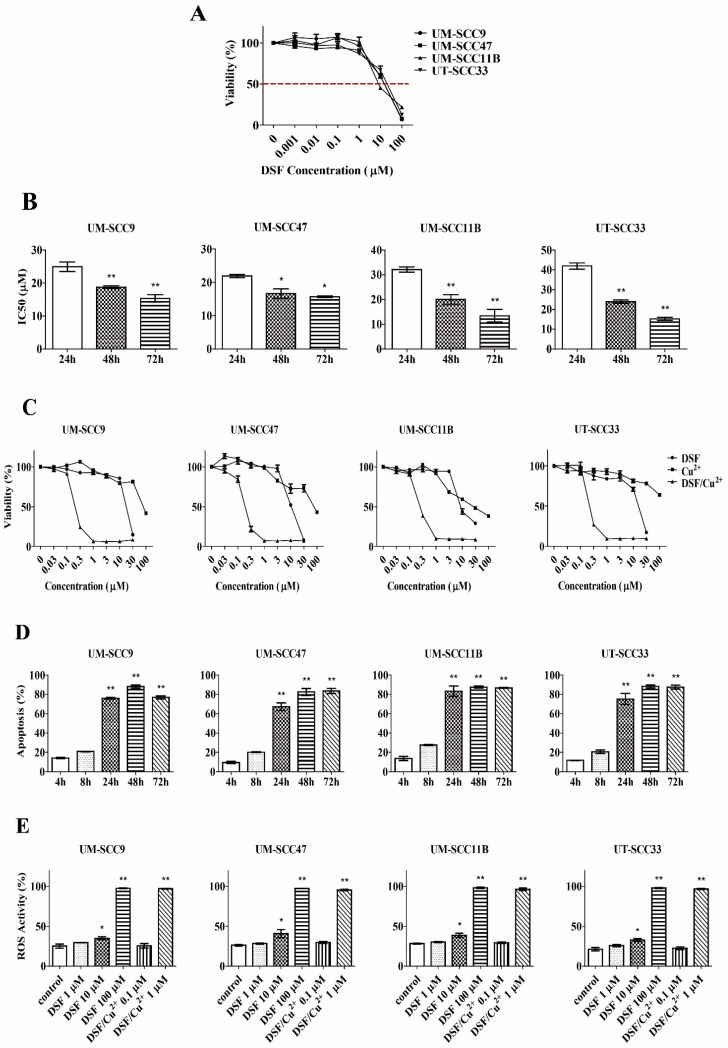
Cytotoxicity and reactive oxygen species (ROS) generation of disulfiram (DSF) or DSF/Cu^2+^. (**A**) Cells were exposed to different concentrations of DSF for 72 h and subjected to the 1-(4,5-dimethylthiazol-2-yl)-3,5-diphenylformazan (MTT) assay, *n* = 3. (**B**) Cells were exposed to different concentrations of DSF (0.1–30 μM) at the indicated time intervals. The levels of IC_50_ were measured by the MTT assay. * *p* < 0.05 and ** *p* < 0.01, *n* = 3, one-way ANOVA, compared to 24 h. (**C**) Cells were exposed to different treatments for 72 h, and the viability was measured by the MTT assay, *n* = 3. (**D**) Cells were exposed to DSF/Cu^2+^ (1 µM) at the indicated time intervals. The apoptotic cell population was identified by the Annexin/V-PI (propidium iodide) assay. ** *p* < 0.01, *n* = 2, one-way ANOVA, compared to 4 h. (**E**) Cells were exposed to different concentrations of DSF or DSF/Cu^2+^ for 24 h; then, the ROS activity was measured. * *p* < 0.05 and ** *p* < 0.01, *n* = 2, one-way ANOVA, compared to the control.

UM-SCC9, UM-SCC47, UM-SCC11B, and UT-SCC33 were treated with DSF in a range of concentrations (0.001–100 μM) for 72 h. As shown in Figure 1A, the viability of the cells was significantly reduced after exposure to DSF in a dose-dependent manner. The cytotoxicity increased linearly with the increasing DSF concentrations in all four tested cell lines. The IC_50_ (Inhibitory concentration 50) values were 13.96 μM, 13.43 μM, 11.24 μM, and 15.06 μM for the cell lines UM-SCC9, UM-SCC47, UM-SCC11B, and UT-SCC33, respectively.

The cytotoxic effect exerted by DSF was also dependent on the time of exposure. When exposed to different concentrations of DSF (0.1–30 μM) for 24 h, 48 h, and 72 h, the average IC_50_ values of DSF in UM-SCC9 were 24.94, 18.74, and 15.32 μM; 21.91, 16.62, and 15.69 μM in UM-SCC47; 32.10, 20.05, and 14.43 μM in UM-SCC11B; and 41.95, 23.89, and 15.19 μM in UT-SCC33, respectively (Figure 1B). The IC_50_ value at 24 h in each of the cell lines was significantly higher than that at 48 h or 72 h. Altogether; these results confirm that the cytotoxic effects of DSF in HNSCC cell lines shows a dose- and time-dependent effect.

### 3.2. Copper (II) Significantly Increases DSF-Mediated Cytotoxicity in a Dose- and Time-Dependent Manner

Even though DSF alone had no significant cytotoxic effect at concentrations lower than 1 μM (Figure 1A), its cytotoxicity was markedly enhanced when the medium was supplemented with copper (II). As shown in Figure 1C, compared to Cu^2+^ or DSF alone, combined DSF/Cu^2+^ exposure was highly cytotoxic, with an IC_50_ of 0.24 μM, 0.193 μM, 0.267 μM, and 0.27 μM in UM-SCC9, UM-SCC47, UM-SCC11B, and in UT-SCC33, respectively. The IC_50_ value of the DSF/Cu^2+^ treatment over 72 h is approximately 50-fold lower than that of DSF alone.

Next, the apoptosis induced by DSF/Cu^2+^ treatment was evaluated. An Annexin-V/PI dual-staining apoptosis assay was performed following an up to 72-h incubation. As shown in Figure 1D and Supplementary Figure S1, both the proportion of early (Annexin-V^+^/PI^−^, lower/right quadrant) and in late apoptosis (Annexin-V^+^/PI^+^, upper/right quadrant) increased during the course of the treatments. A substantial increase of cytotoxicity was detected following a 24-h or longer incubation. We thus conclude that DSF and Cu^2+^ in combination are remarkably more cytotoxic than their individual components. Nevertheless, the effect is time-dependent. 

### 3.3. ROS Activity Mediates DSF or DSF/Cu^2+^-Induced Cytotoxicity

To assess whether DSF or DSF/Cu^2+^-induced apoptosis is mediated by intracellular ROS formation, the ROS activity was measured with increasing concentrations of DSF (1–100 μM) or DSF/Cu^2+^ (0.1–1 μM). As shown in Figure 1E and Supplementary Figure S3, The ROS activity intensified with the increasing concentrations of DSF or DSF/Cu^2+^. More ROS was generated when the DSF concentration was elevated from 10 μM (33.1%, 35.1%, 36.9%, and 32.6%) to 100 μM (98.1%, 97.6%, 97.9%, and 98.5% in UM-SCC9, UM-SCC47, UM-SCC11B, and UT-SCC33, respectively). Importantly, the combination of DSF (1 μM) and Cu^2+^ (1 μM) resulted in a strikingly enhanced ROS accumulation; the effect was similar to that observed in cells treated with 100 μM of DSF alone. These findings suggest that DSF or DSF/Cu^2+^-induced cytotoxicity is mediated by the generation of intracellular ROS. Moreover, the addition of Cu^2+^ enhances ROS production about 100-fold compared to DSF alone.

### 3.4. DSF or DSF/Cu^2+^ Does Not Interfere with Cell Cycle Progression

To analyze the mechanisms underlying the cytotoxic effects of DSF or DSF/Cu^2+^, the cell cycle progression was investigated. Since the cell cycle phases were compromised and disappeared at high drug concentrations, the concentrations used for this assay were selected for each cell line based on the IC_50_ values to avoid excessive apoptosis during exposure. As shown in Supplementary Figure S2, cells were treated with different concentrations of DSF (0.1–3 μM) or DSF/Cu^2+^ (0.01–0.1 μM). The proportions of cells in the G1, S, and G2/M phases did not significantly change compared to the untreated controls. These results indicate that either DSF alone or in combination with Cu^2+^ does not affect the cell cycle progression of HNSCC cell lines under the experimental conditions used.

### 3.5. DSF Enhances Cisplatin Cytotoxicity

**Figure 2:**
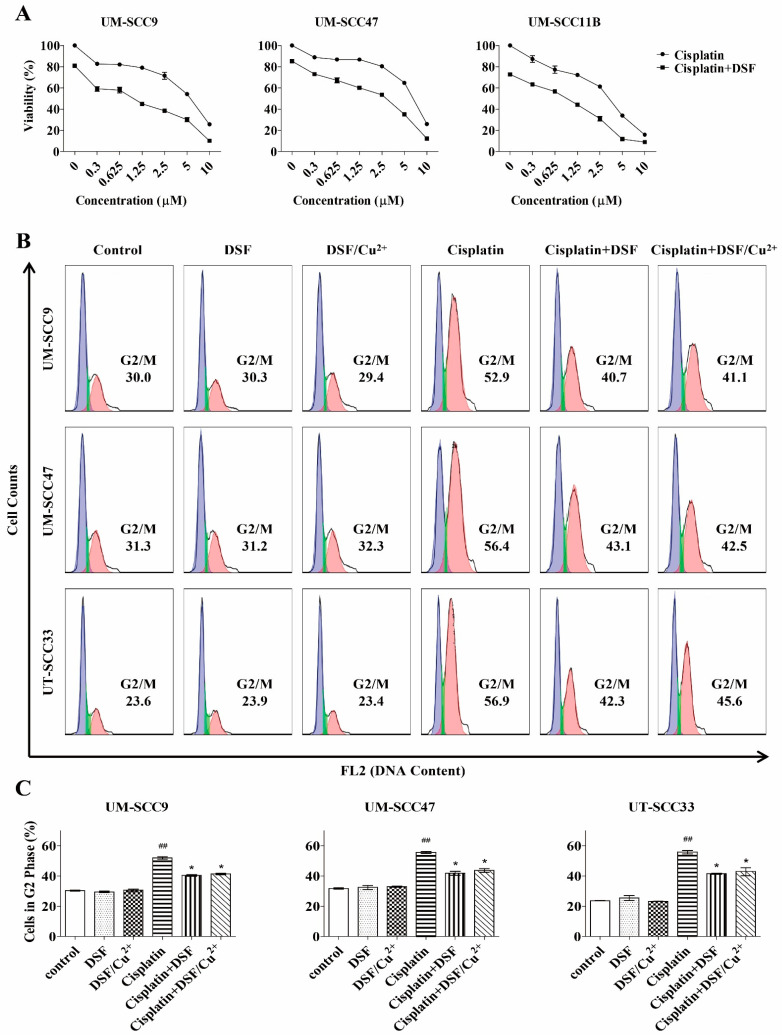
Combination treatment with DSF or DSF/Cu^2+^ and cisplatin. (**A**) Cells were exposed to different concentrations of cisplatin with DSF (5 µM) for 72 h, and the cytotoxicity was measured by the MTT assay, *n* = 3. (**B**) Cells were exposed to DSF (5 µM), DSF/Cu^2+^ (0.1 µM), cisplatin (UM-SCC9 and UM-SCC47: 0.3 µM and UT-SCC33: 0.6 µM), or a combination of both for 48 h. (**C**) The percentage distribution of cells in the G2/M phase is compared. ^##^ *p* < 0.01: cisplatin vs. control and * *p* < 0.05: cisplatin vs. cisplatin + DSF or cisplatin + DSF/Cu^2+^, *n* = 2, one-way ANOVA.

We further investigated whether DSF and cisplatin have an impact compound in the treatment. The cytotoxicity of DSF (5 μM) and cisplatin in combination was significantly higher than that of cisplatin alone (Figure 2A). To understand the essence of this combinatorial effect, the Chou-Talalay method was used to distinguish different interactions, as described in Methods. As shown in Table 1, the combination of DSF and cisplatin yielded a synergistic effect (CI < 1) in all four tested cell lines in a broad concentration at ED_50_ and ED_75_ (effective dose). These observations provide strong evidence that DSF synergistically reduces cell proliferation and enhances the cytotoxicity of cisplatin.

### 3.6. DSF or DSF/Cu^2+^ Inhibits Cisplatin-Induced G2/M Phase Arrest

To gain a better understanding of the cytotoxic effects induced by DSF or DSF/Cu^2+^, the cell cycle progression was assessed following a 48-h treatment with cisplatin in the presence of DSF or DSF/Cu^2+^. As seen in Figure 2B,C, cisplatin enhanced the G2/M blockade, which resulted in an increased number of cells in the G2/M phase and in a reduced number of cells in G1/G0 as well. A highly pronounced G2/M phase arrest was seen in the three tested cell lines (30.0–52.9% in UM-SCC9, 31.3–56.4% in UM-SCC47, and 23.6–56.9% in UT-SCC33). However, when the cells were treated with cisplatin in combination with 5 μM of DSF or 0.1 μM of DSF/Cu^2+^, the number of cells in the G2/M populations decreased, with a parallel increase in the number of cells in the G1 phase. A significant abrogation was observed from 52.9% to 40.7% and 41.1% in UM-SCC9, from 56.4% to 43.1% and 42.5% in UM-SCC47, and from 56.9% to 42.3% and 45.6% in UT-SCC33. Collectively, DSF or DSF/Cu^2+^ partly reverse this cisplatin-induced blocking and resulted in a lower proportion of cells in the G2/M phase. 

### 3.7. Radio-Sensitizing Effects of DSF or DSF/Cu^2+^

**Figure 3:**
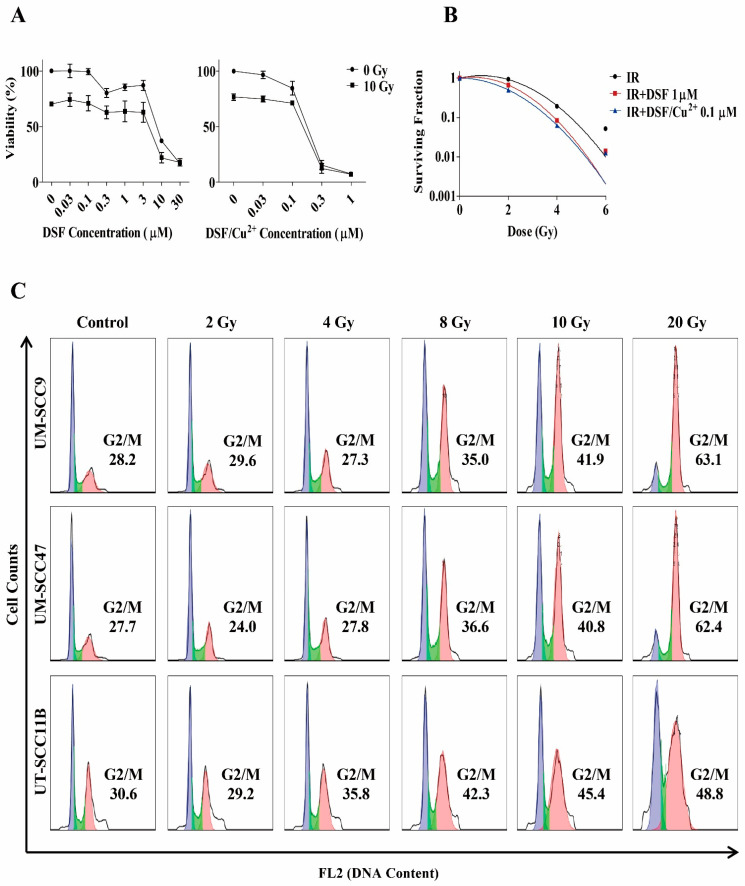

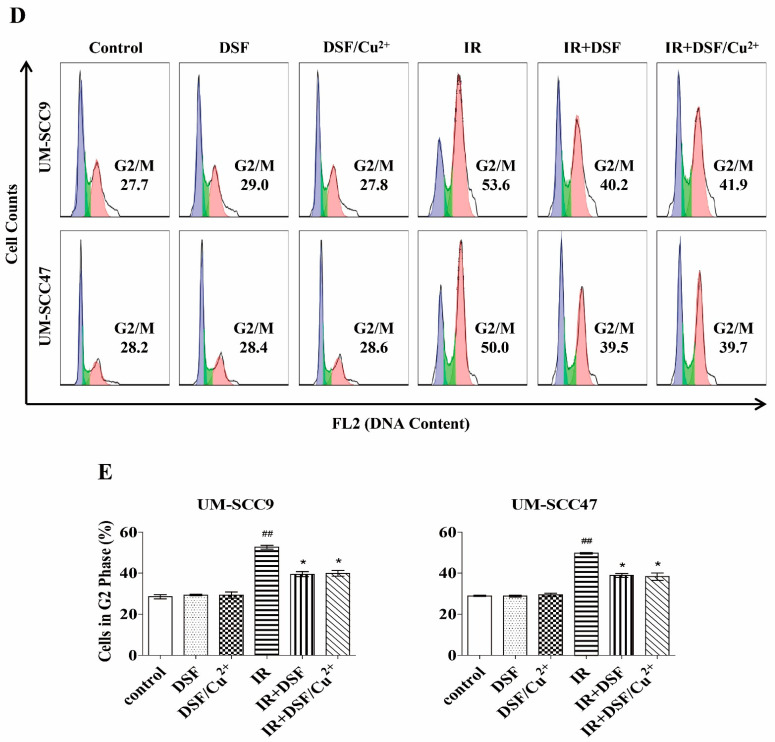
Radio-sensitizing effect of DSF or DSF/Cu^2+^. (**A**) Cells were pretreated with different concentrations of DSF or DSF/Cu^2+^ and then irradiated with 10 Gy. Viability was measured by the MTT assay after 72 h, *n* = 3. (**B**) Cells were pretreated with DSF (1 µM) or DSF/Cu^2+^ (0.1 µM) and then irradiated with 2–6 Gy. After 24 h, cells were reseeded in the drug-free medium for 9–12 days. The surviving fraction was compared using the formula of the linear-quadratic (LQ) model. (**C**) Cells were exposed to different dosages of IR, and then, the cell cycle distribution was measured 48 h later. (**D**) Cells were pretreated with DSF (5 µM) or DSF/Cu^2+^ (0.1 µM) before being exposed to IR (10 Gy). After recovering for a further 48 h, the levels of each cell cycle phase were determined. (**E**) The percentage of cells in the G2/M phase was compared. ^##^ *p* < 0.01: IR vs. control and * *p* < 0.05: IR vs. IR + DSF or IR + DSF/Cu^2+^, *n* = 2, one-way ANOVA.

To determine whether DSF or DSF/Cu^2+^ increase the sensitivity of HNSCC cells to IR, cells were pretreated with increasing concentrations of DSF or DSF/Cu^2+^, then exposed to IR (10 Gy) and rested for 72 h. Dose response curves showed an increased cytotoxicity when DSF or DSF/Cu^2+^ were combined with IR (Figure 3A). 

To investigate whether DSF or DSF/Cu^2+^ could exhibit a radio-sensitizing effect, we incubated cells with DSF (1 μM) or DSF/Cu^2+^ (0.1 μM) before exposing them to a graded dosage of IR. Cell survival following IR was shown in Figure 3B. DSF and DSF/Cu^2+^ strongly inhibited HNSCC clonogenicity to such an extent that few clones could be isolated and counted to establish the curve with this combination therapy. Taken together, these findings demonstrate both DSF and DSF/Cu^2+^ significantly increased the cytotoxic effects of IR in the HNSCC cell lines.

### 3.8. Treatment with DSF or DSF/Cu^2+^ Reduces IR-Induced G2/M Phase Arrest

To explore the potential mechanism underlying the radio-sensitizing effect of DSF or DSF/Cu^2+^, we analyzed their impact on IR-induced changes in the cell cycle progression. First, we measured the cell cycle status when cells were exposed to increasing dosages of IR (Figure 3C). After a relatively high dose, such as 20 Gy, most cells were blocked in the G2/M phase (63.1% in UM-SCC9, 62.4% in UM-SCC47, and 48.8% in UM-SCC11B). 

To determine whether DSF or DSF/Cu^2+^ reactivates the cell cycle progression through G2/M and therefore potentially sensitizes cells to IR, DSF (5 μM) or DSF/Cu^2+^ (0.1 μM) was added to the culture of UN-SCC9 and UM-SCC47 cell lines prior to IR (Figure 3D,E). At 48 h post-IR, an activation of G2/M arrest occurred from 27.7% to 53.6% in UM-SCC9 and from 28.2% to 50.0% in UM-SCC47, with a concomitant drop in the number of cells in the G1 phase. In contrast, in cells pretreated with 5-μM DSF or 0.1-μM DSF/Cu^2+^, IR resulted in a decreased fraction in the G2/M populations from 53.6% to 40.2% and 41.9% in UMS-CC9 and from 50.0% to 39.5% and 39.7% in UM-SCC47. In conclusion, these observations suggest that a pretreatment with DSF or DSF/Cu^2+^ attenuate the IR-induced G2/M phase arrest.

### 3.9. Cytotoxicity of the Combined Treatment with DSF or DSF/Cu^2+^, Cisplatin, and IR

**Figure 4:**
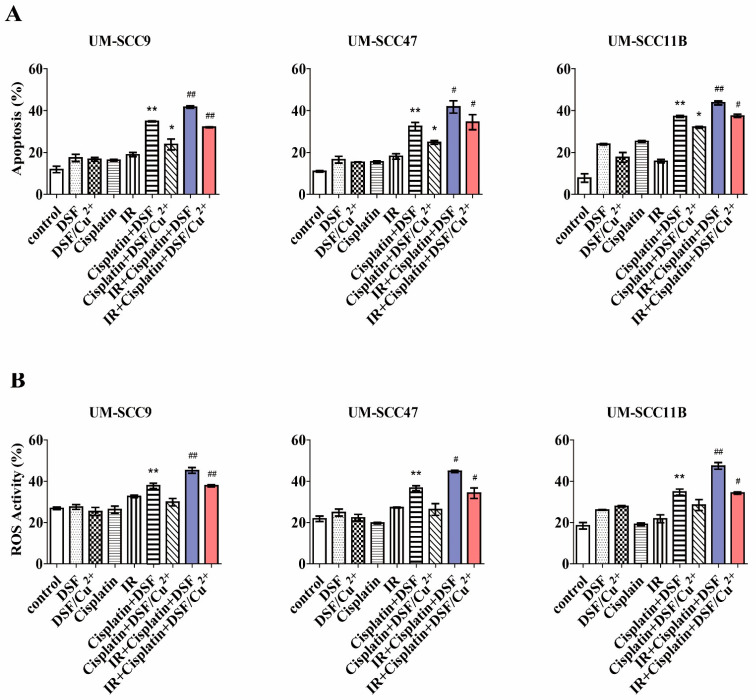
DSF or DSF/Cu^2+^ combined with cisplatin and irradiation (IR) enhances apoptosis and reactivw oxygen species (ROS) generation. Cells were pretreated with DSF (5 µM), DSF/Cu^2+^ (0.1 µM), cisplatin (2.5 µM), or a combination of both and then exposed to IR (10 Gy). (**A**) Apoptosis was determined with Annexin/V-PI staining 48 h later by flow cytometry. ** *p* < 0.01: cispaltin + DSF vs. cisplatin or DSF, * *p* < 0.05: cisplatin + DSF/Cu^2+^ vs. cisplatin or DSF/Cu^2+^, and ^#^ *p* < 0.05 or ^##^ *p* < 0.01: IR + cisplatin + DSF vs. cisplatin + DSF or IR + cisplatin + DSF/Cu^2+^ vs. cisplatin + DSF/Cu^2+^, *n* = 2, one-way ANOVA. (**B**) ROS accumulation was determined after 24 h of treatment by flow cytometry. * *p* < 0.05 or ** *p* < 0.01: cisplatin + DSF vs. cisplatin or DSF and ^#^ *p* < 0.05 or ^##^ *p* < 0.01: IR + cisplatin + DSF vs. cisplatin + DSF or IR + cisplatin + DSF/Cu^2+^ vs. cisplatin + DSF/Cu^2+^, *n* = 2, one-way ANOVA.

The above presented experiments demonstrate that DSF or DSF/Cu^2+^ enhanced the cytotoxicity of cisplatin and IR by reversing the G2/M checkpoint arrest and reducing the colony formation. These results prompted us to investigate the effects of triple treatment by using a combination capacity of DSF or DSF/Cu^2+^, cisplatin, and IR. As shown in Figure 4A and Supplementary Figure S4, DSF (5 μM) or DSF/Cu^2+^ (0.1 μM) in combination with cisplatin (2.5 μM) induced the apoptotic cells 34.71% and 26.69% in UM-SCC9, 31% and 25.48% in UM-SCC47, and 37.55%, and 31.77% in UM-SCC11B. Thus, a treatment with DSF or DSF/Cu^2+^ pushed cisplatin-damaged cells into apoptosis. More importantly, we proved in the previously shown experiments (Figure 2A) that the viability of the cells at 2.5-μM cisplatin was nearly 80%, and here, we also indicated that apoptosis at 2.5-μM cisplatin was lower than 20%. In contrast, when combined with DSF, even at this low concentration of cisplatin, a statistically significant stronger toxicity was induced. In addition, a substantial increase of apoptosis was found when combined with IR. Apoptosis of 42.04% and 32.21% was observed in IR + cisplatin + DSF and IR + cisplatin + DSF/Cu^2+^ in UM-SCC9, 43.9% and 31.91% in UM-SCC47, and 45.37% and 38.08% in UM-SCC11B. This impact of DSF or DSF/Cu^2+^ on the cytotoxicity as seen in the triple-combination treatments emphasizes the potential clinical use of this strategy to counteract chemo-radio-resistance, leading to better therapeutic outcomes.

### 3.10. Combination of DSF, DSF/Cu^2+^, Cisplatin, and IR Enhances ROS Generation

Based on the cytotoxic effect of the triple treatment shown above, we hypothesized that the ROS level may correspond to the apoptotic rate induced by DSF or DSF/Cu^2+^. Compared with each treatment alone, the combined DSF (5 μM) with cisplatin (2.5 μM) increased the ROS levels to 38.7% in UM-SCC9, 35.8% in UM-SCC47, and 35.9% in UM-SCC11B, respectively (Figure 4B and Supplementary Figure S5). IR is also an essential element for ROS generation. When combined with IR, cisplatin and DSF or DSF/Cu^2+^ further increased the ROS activity to 46.3% and 37.4% in UM-SCC9, 44.0% and 32.5% in UM-SCC47, and 46.3% and 34.7% in UM-SCC11B, respectively. Taken together, these results indicate that a triple treatment of DSF or DSF/Cu^2+^ with cisplatin and IR significantly increases the cytotoxicity in the HNSCC cell lines by enhancing the ROS accumulation.

### 3.11. DSF or DSF/Cu^2+^ Inhibits Spheroid Formation

**Figure 5:**
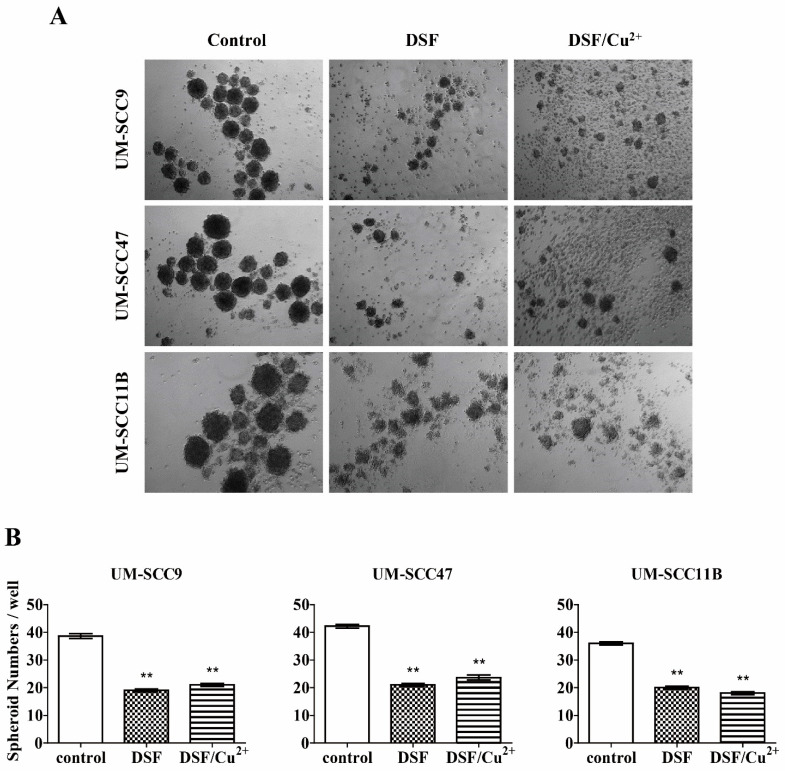
DSF or DSF/Cu^2+^ inhibits spheroid formation in the head and neck squamous cell carcinoma (HNSCC) cell lines. (**A**) Cells were exposed to DSF or DSF/Cu^2+^ for 72 h, and representative images are shown (×50 magnification). (**B**) Histogram shows the statistical analysis of spheroid numbers. ** *p* < 0.01 one-way ANOVA compared to control.

To evaluate the stemness, epithelial cells from stablished cells lines were investigated for their ability to grow independently in an anchorage. This spheroid formation assay was performed to measure the activity of stem cells and to investigate the potential effect of the DSF or DSF/Cu^2+^ treatment on the self-renewal capacity of the HNSCC cell lines. As shown in Figure 4A,B, an abundance of large spheroids grew from untreated controls in all three tested cell lines. We observed small inattentive spheroids and loose cellular aggregates when cells were exposed to DSF (10 μM) or DSF/Cu^2+^ (0.15 μM) for three days, and the ability of the spheroid formation was remarkably reduced, which indicated a reduced proliferative potential of the cancer stem cells (CSCs) (Figure 5). The number of spheroids was significantly reduced from an average of 39 to 19 and 21 in UM-SCC9, from an average of 42 to 21 and 24 in UM-SCC47, and from an average of 36 to 20 and 18 in UM-SCC11B, respectively. In conclusion, a DSF or DSF/Cu^2+^ treatment could inhibit the formation and growth of spheroids, indicating their possible inhibition of self-renewal in HNSCC CSCs.

### 3.12. DSF Induced Growth Inhibition of HNSCC-Derived PDX Models

**Figure 6:**
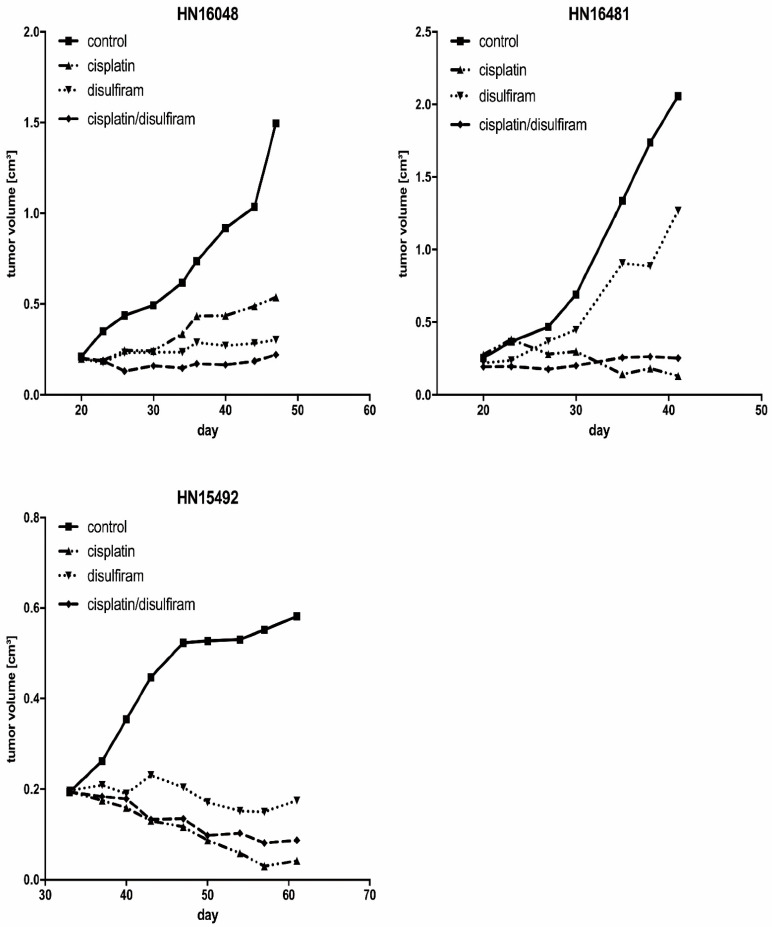
DSF induced the growth inhibition in HNSCC-derived patient-derived tumor xenograft (PDX) models. Three independently derived permanently growing PDX models from a primary HNSCC tumor were transplanted onto severe combined immune deficiency (SCID) mice and single animals, each treated by DSF, cisplatin, or a combination. Tumor volumes were measured twice a week.

For further evaluation of the DSF effects in vivo, we propagated three different HNSCC PDX models and treated the mice with disulfiram and cisplatin separately and in combination in a pilot trial to support the feasibility murine model using HNSCC xenotransplants. These results confirm an effective oral application route resulting in sufficient (tumor) tissue levels for a treatment evaluation with cisplatin-based chemotherapy and radiation. As shown in Figure 6, compared to the controls, mice were treated with DSF alone or in combination with cisplatin to reach the therapeutic levels in mice and at the tumor site. Future testing of our in vitro data for statistical significance of additives or synergistic effects using combination therapies is warranted.

## 4. Discussion

The current treatment strategies for HNSCC are surgery combined with radiation or chemotherapy. However, approximately 50% of patients who are initially treated will develop therapy-resistant recurrent tumors within two years after treatment, providing a poor prognosis [27,28]. The development of effective strategies to counteract resistance, reduce toxicity, and to improve clinical outcome are urgently needed. Our study demonstrates the high potential of DSF or DSF/Cu^2+^ as a chemo-radio-sensitizing agent for HNSCC in vitro. We identified that the viability of HNSCC cells was inhibited by DSF, and the addition of Cu^2+^ further enhanced this cytotoxicity in a dose- and time-dependent manner. These observations were consistent with previous findings that DSF has anticancer activity and that copper potentiates its activity in vitro and in vivo [9,14]. 

In particular, our findings showed that DSF synergistically reduced the cellular proliferation and enhanced the cisplatin-induced cytotoxicity. Cisplatin is a platinum-based compound that forms both intra- and inter-strand DNA adducts that have a broad spectrum of antitumor activity and are widely used in the treatment of various malignant tumors [29]. Although it has long been used throughout history, a successful cisplatin treatment has two major obstacles—severe toxicity and acquired resistance [30]. It is well-known that a cellular sensitivity to chemotherapeutic agents relies on DNA damage and deregulated cell cycle kinetics allowing damaged cells to undergo mitosis, leading to cell death [31]. Cisplatin is a potent cytotoxic agent that induces cell death by directly damaging DNA, which covalently binds DNA to form bulky adducts that block replication and transcription, resulting in G2 phase cell cycle arrest [32]. Attenuation of the G2 checkpoint and subsequently enhanced cytotoxicity nicely fits with the rationale that unrepaired DNA damage will eventually lead to a loss of clonogenicity and result in cell death [20]. We found that the cell cycle was blocked at G2/M when the cells were exposed to cisplatin, and an additional treatment by DSF or DSF/Cu^2+^ could partially reverse this blocking. Our results thus indicate that DSF or DSF/Cu^2+^ may prevent G2/M cell cycle checkpoint activation and, thus, sensitize the tumor cells to cisplatin treatment.

We observed that, after treatment with IR, the G2/M cell fractions were increased in a dosage-dependent manner, which suggests a greater checkpoint activation in response to DNA damage. We further demonstrated that DSF or DSF/Cu^2+^ potentiated the efficacy of IR through attenuating the G2/M arrest to inhibit IR-induced G2 checkpoint activation, which could enable more damaged cells to enter mitosis without appropriate repair for a further accumulation of DNA damage and thereby significantly enhance the cytotoxic efficacy of IR. This hypothesis may constitute a potential mechanism underlying the cisplatin- or IR-enhancing effects of DSF or DSF/Cu^2+^ in HNSCC cell lines. 

IR induces DNA double-strand breaks and typically activates the G2/M checkpoint, which arrests cells in the G2/M phase. This arrest provides time to repair DNA damage and to prevent mitotic catastrophes and apoptosis [33]. Abrogation of the G2/M checkpoint has been regarded as a clinically exploitable strategy to achieve tumor-specific radio-sensitization [34]. In particular, critical thiols in effectors of DNA repair, the cell cycle checkpoint, and other pathways linked to the cellular response to IR are possible targets for DSF or DSF/Cu^2+^-induced radio-sensitization [35]. Many studies have shown that abrogation of the G2 checkpoint can potentiate cell death induced by IR [36] or DNA-damaging agents [37], which supports the G2 checkpoint as a potential therapeutic target that may sensitize cells to chemo- and radiotherapy. Moreover, a DSF or DSF/Cu^2+^ treatment strongly decreased the clonogenicity and, in conjunction with IR, resulted in significantly fewer clones isolated and counted. Those findings indicate that DSF or DSF/Cu^2+^ could be used to synergize with IR in a combination therapy.

Our data demonstrated that DSF or DSF/Cu^2+^ alone or in combination with cisplatin and IR significantly increased the intracellular ROS accumulation, which further supported DSF as a novel combination treatment regimen for HNSCC treatment. ROS contains a group of oxygen-containing chemical species normally generated from the mitochondrial respiratory chain reaction with reactive chemical properties [38]. ROS affects cellular processes such as proliferation, senescence, and apoptosis that contribute to the development of cancer [39]. Owing to the high proliferative rate and energy requirements, cancer cells are under higher ROS stress than their normal counterparts. High levels of ROS can damage DNA, the mitochondrial inner membrane, and membrane phospholipids, leading to apoptosis [40]. It has been demonstrated that a further exposure of ROS induced by ROS-generating agents (such as DSF or DSF/Cu^2+^) could exhaust the cellular antioxidant capacity, pushing tumor cells over the tolerated ROS threshold and selectively leading to apoptosis in tumor cells [41]. Interestingly, the stemness of the established HNSCC cell lines was significantly reduced after treatment with DSF or DSF/Cu^2+^. We suppose that cancer stem cells that are characterized by high levels of ALDH expression are especially vulnerable to DSF treatment and ROS generation. This, in turn, could lead to reduced tumor growth capacity and less therapy resistance.

Our study further implicated DSF as a potential drug targeting HNSCC. In recently established PDX models from HNSCC primary tumors, the DSF treatment showed a remarkable growth retardation that was significant in two out of three independent models. This effect was retained in all combination therapy models with cisplatin demonstrating both the sufficient tumor tissue levels of DSF and cisplatin alone in vivo and in combination for tumor treatment evaluation, bearing in mind that the observed induction of apoptosis by DSF in vitro is important for the design of murine in vivo trials. However, due to the cisplatin sensitivity and the limited number of mice used, no synergistic effect could be demonstrated at this point. These data, although generated in an initial pilot trial, already show promise for an applicability of DSF for HNSCC therapy. A study evaluating combination treatment regimens statistically will be needed for characterization of the therapeutic potential in preparation of clinical trials.

Indeed, the anticancer activity of DSF appears promising in clinical trials in various malignancies. An initial assessment of DSF in combination with cisplatin in non-small cell lung cancer was recently completed (NCT 00312819). The results indicated that patients receiving additional DSF treatment showed increased survival. A phase II trial is investigating the effect of DSF in a newly diagnosed glioblastoma multiform (NCT 01777919). Other phase I clinical trials of DSF in refractory solid tumors, including liver (NCT 00742911), melanoma metastases (NCT 00256230), and prostate cancer (NCT 01118741), are still undergoing. The development of a novel anticancer drug against various malignant tumors is both a time-consuming and costly procedure. Finding a novel therapeutic use for an “old” drug has attracted attention, owing to being fast tracked, and may lead to the discovery of new biological processes or disease pathways [42]. DSF has been an FDA-approved drug to treat alcoholism for over 60 years, since 1951. It could be a promising strategy for repositioning it as a prospective drug or adjuvant treatment for sensitization in combination with other chemo or radio treatments to overcome resistances to single compounds. Even though the impressive physiological tolerance of DSF and its powerful anticancer effects has caught the attention of researchers for years, the development of DSF-based clinical applications against tumor diseases still needs to move forward, including for HNSCC. 

The obstacles in this path are the instability of DSF in the gastric environment and its rapid degradation by glutathione reductase, which appears to cause unfavorable pharmacokinetics and pharmacodynamics, hampering its further development as an anticancer drug [43]. Clinical studies have demonstrated that, after a single oral dose of 500 mg or repeated doses of 250 mg, the plasma concentrations of DSF were less than 2 μM [17]. Therefore, an efficient drug delivery system is essential for the clinical application of DSF in cancer treatment. One strategy would be the encapsulation of DSF into nanoparticles to protect it from degradation in the blood system. The targeted delivery of DSF-encapsulated nanoparticles into tumor cells could increase the drug accumulation at the tumor site and would enhance the endurance of the drug in the blood circulation [9,44]. Due to the intrinsic Cu^2+^ distribution inside the cells and the ROS modulative property, a low dosage of DSF could already oxidize the cancer cells to death, especially when oxidative stress is imposed. This implies that a higher concentration may not be necessary for cancer therapy. Instead, proper doses of DSF matched with Cu^2+^ are more important. Therefore, the ROS-independent mechanism is expected to be rather important at clinically achievable concentrations, and the carefully controlled titration of DSF and its derivatives for cancer treatment remain to be investigated.

In summary, we demonstrated that DSF or DSF/Cu^2+^ could induce cytotoxicity in a dose- and time-dependent manner and that the cytotoxic effect was mediated by ROS generation. Further, we showed that DSF or DSF/Cu^2+^ sensitized HNSCC cells towards IR and the cisplatin treatment effectively and partially reversed the protective G2/M phase cell cycle arrest. Due to its intrinsic cytotoxic and radio-chemo-sensitizing effects, DSF or DSF/Cu^2+^ could be used as a radio-chemo sensitizer to overcome the therapeutic resistance and, thus, improve the successful treatment of HNSCC in the future.

## Figures and Tables

**Table 1:** Disulfiram (DSF) and cisplatin shows synergistic cytotoxicity.

Cisplatin + DSF	Combination Index (CI) ^a^ at	
	ED_50_	ED_75_
UM-SCC 9	0.560	0.447
UM-SCC47	0.676	0.624
UM-SCC11B	0.543	0.551
UT-SCC33	0.645	0.507

^a^ CI for DSF plus cisplatin compared to individual application. CI = 1 indicates an additive effect, CI < 1 indicates a synergistic effect, and CI > 1 indicates an antagonistic effect.

## Data Availability

The data presented in this study are available on request from the corresponding author. The data are not publicly available due to privacy.

## Supplementary Materials

The following are available online at https://www.mdpi.com/2073-4409/10/3/517/s1.
