# Supplementary material for: Disulfiram Acts as a Potent Radio-Chemo Sensitizer in Head and Neck Squamous Cell Carcinoma Cell Lines and Transplanted Xenografts
Source: Cells. 2021 Feb 28;10(3):517. doi: 10.3390/cells10030517 (PMC7999545; doi:10.3390/cells10030517)
Supplement: Supplementary file 1 [file cells-10-00517-s001.zip › Supplementary Files/Supplementary Results.docx]

**Supplementary Results**

**Figure S1: DSF/Cu^2+^ induces apoptosis in a time-dependent manner.**

Cells were exposed to DSF/Cu^2+^ (1 µM/1 µM) for indicated time intervals. The percentage of the different cell populations discriminated by Annexin-V assay is given in each quadrant (Q). The Annexin-V^+^ populations (upper-right and lower-right) represent apoptotic cells. A substantial increase in apoptotic cells was detected after exposure for 24 h.

**Figure S2: DSF or DSF/Cu^2+^ does not interfere with cell cycle progression.**

The DNA content distribution of cells after treatment with DSF or DSF/Cu^2+^ for 72 h was analyzed by flow cytometry. Numbers in the graph represent proportions in percent of G1, S and G2/M phase.

**Figure S3: DSF or DSF/Cu^2+^ increases the ROS generation.**

Cells were exposed to different concentrations of DSF or DSF/Cu^2+^ for 24 h, and then the ROS activity was measured by flow cytometry. Numbers in the graph represent ROS activity. Combination of DSF (1 μM) and Cu^2+^ (1 μM) result in a striking increase in intracellular ROS accumulations, which is equivalent to the effect of 100 μM DSF alone.

**Figure S4: Cytotoxicity of the combined treatment with DSF or DSF/Cu^2+^, cisplatin and IR.**

The cancer cells were pre-treated with DSF (5 µM), DSF/Cu^2+^ (0.1 µM), cisplatin (2.5 µM), or a combination of both, then exposed to IR (10 Gy). 48 h later, cells were stained with Annexin/V-PI. The percentage of the different cell populations discriminated is given in each quadrant (Q). The Annexin-V^+^ populations (upper-right and lower-right) represent apoptotic cells.

**Figure S5: Combination of DSF or DSF/Cu^2+^, cisplatin and IR enhances ROS generation.**

The cancer cells were pre-treated with DSF (5 µM), DSF/Cu^2+^ (0.1 µM), cisplatin (2.5 µM), or a combination of both, then exposed to IR (10 Gy). 24 h later, ROS activity was measured by flow cytometry. Numbers in the graph represent the ROS activity.
