# Supplementary figures and images for: Disulfiram Acts as a Potent Radio-Chemo Sensitizer in Head and Neck Squamous Cell Carcinoma Cell Lines and Transplanted Xenografts
Source: Cells. 2021 Feb 28;10(3):517. doi: 10.3390/cells10030517 (PMC7999545; doi:10.3390/cells10030517)

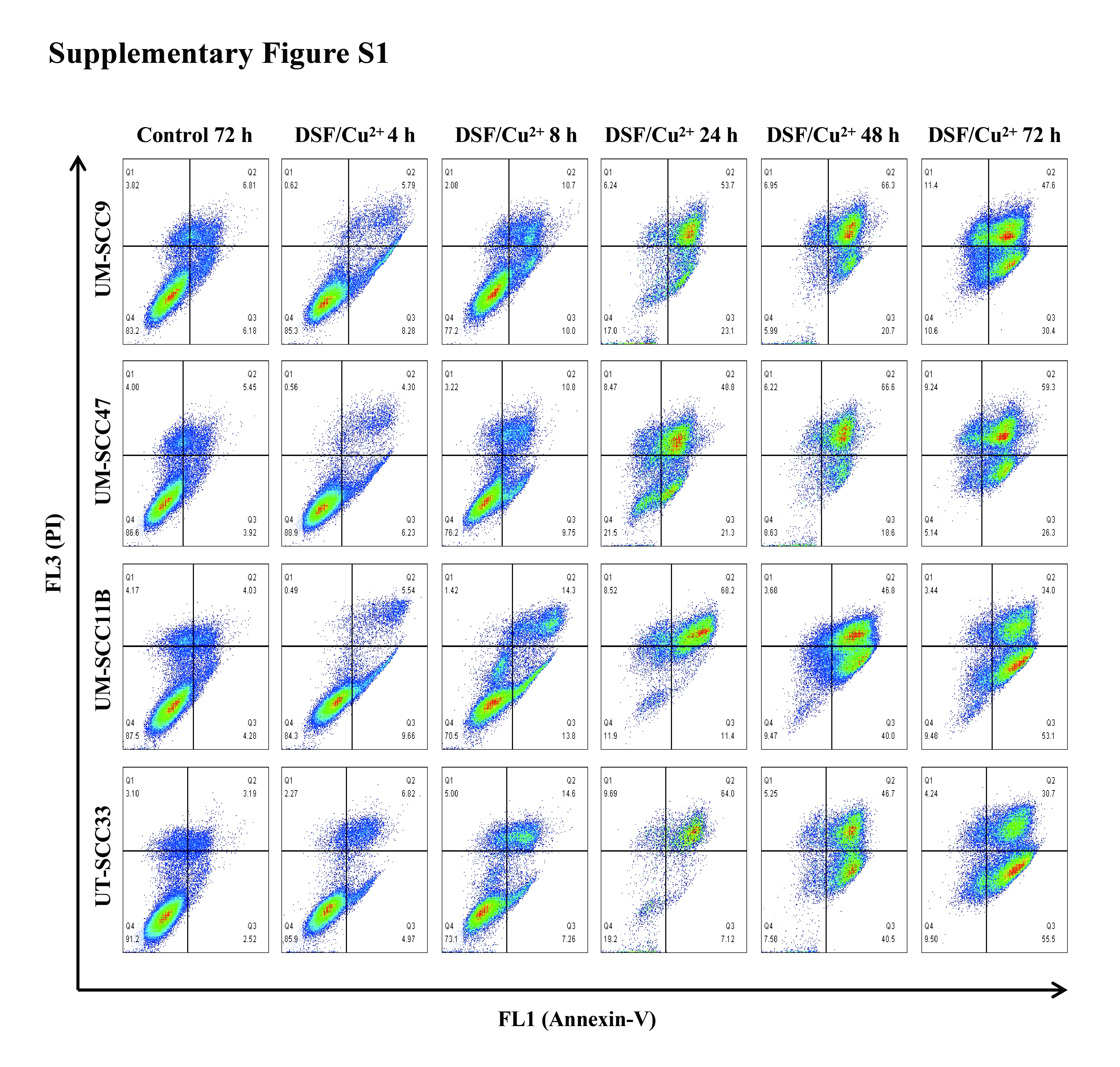

Supplement: Supplementary file 1 [file cells-10-00517-s001.zip › Supplementary Files/Supplementart Figure S1.jpg]

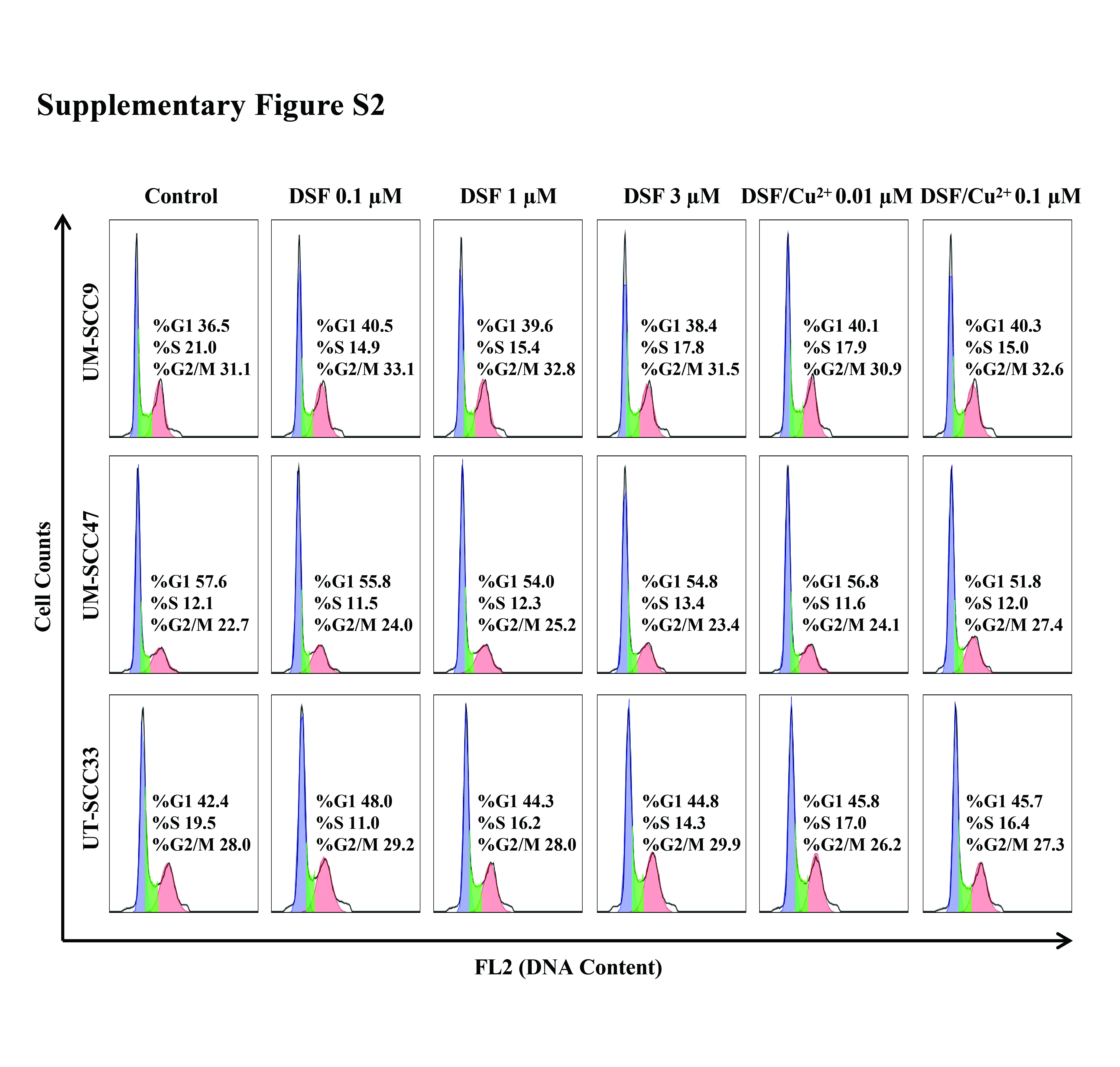

Supplement: Supplementary file 1 [file cells-10-00517-s001.zip › Supplementary Files/Supplementary Figure S2.jpg]

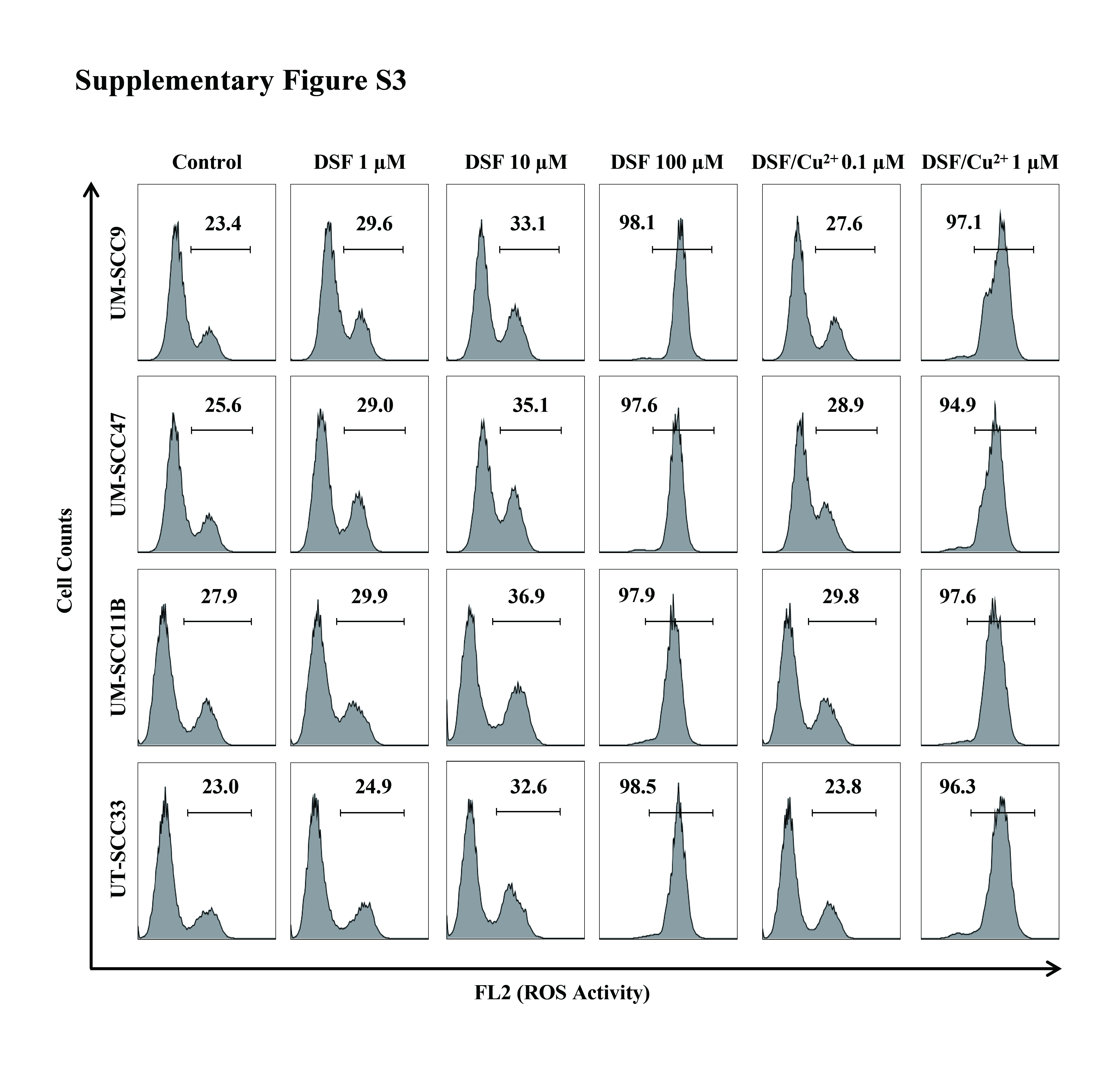

Supplement: Supplementary file 1 [file cells-10-00517-s001.zip › Supplementary Files/Supplementary Figure S3.jpg]

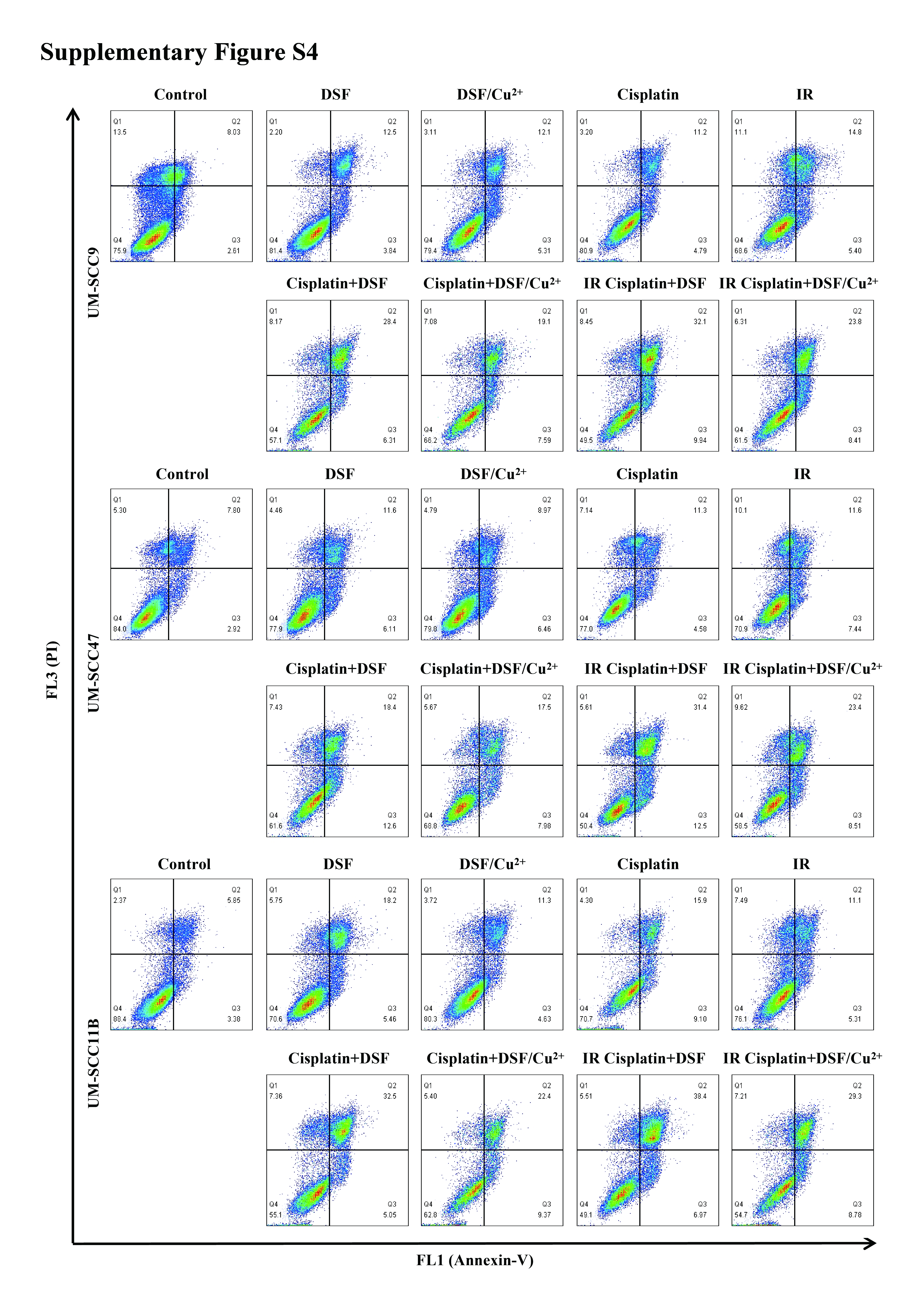

Supplement: Supplementary file 1 [file cells-10-00517-s001.zip › Supplementary Files/Supplementary Figure S4.jpg]

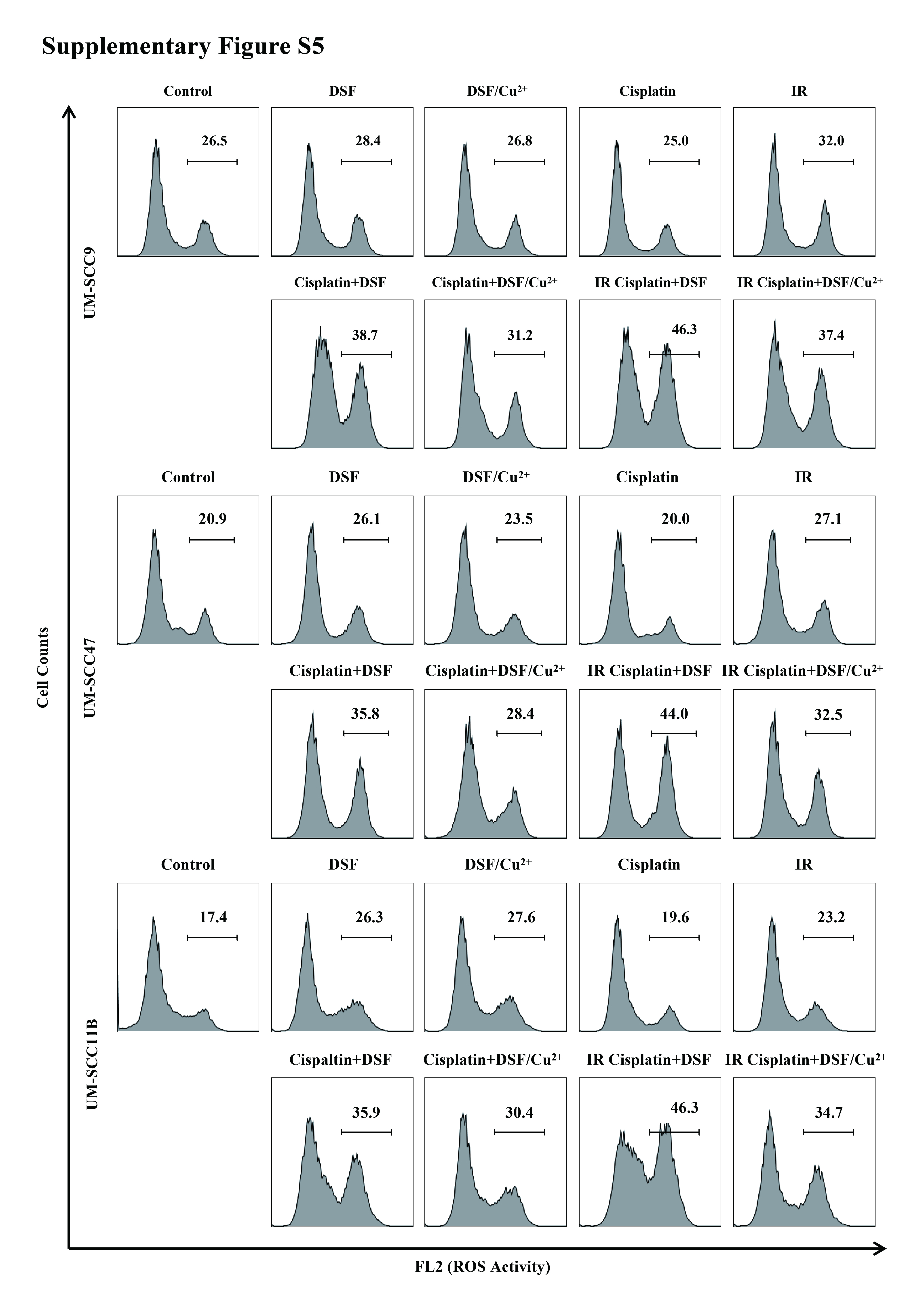

Supplement: Supplementary file 1 [file cells-10-00517-s001.zip › Supplementary Files/Supplementary Figure S5.jpg]
